# Adavosertib (AZD1775) does not prolong the QTc interval in patients with advanced solid tumors: a phase I open-label study

**DOI:** 10.1007/s00280-023-04555-2

**Published:** 2023-06-27

**Authors:** Mats Någård, Mei-Lin Ah-See, James Strauss, Trisha Wise-Draper, Howard P. Safran, Laura Nadeau, William J. Edenfield, Lionel D. Lewis, Dinko Rekić, Corina Dota, Lone H. Ottesen, Yan Li, Ganesh M. Mugundu

**Affiliations:** 1 Clinical Pharmacology and Quantitative Pharmacology, Clinical Pharmacology and Safety Sciences, AstraZeneca, One MedImmune Way, Gaithersburg, MD 20878 USA; 2 Late Stage Development, Oncology R&D, AstraZeneca, Cambridge, UK; 3 Mary Crowley Cancer Research Center, Dallas, TX USA; 4 University of Cincinnati Cancer Center, Cincinnati, OH USA; 5 Rhode Island Hospital, Lifespan Cancer Institute, Providence, RI USA; 6 Beaumont Cancer Center, Royal Oak, MI USA; 7 Institute for Translational Oncology Research, Greenville, SC USA; 8 The Dartmouth Cancer Center and Dartmouth-Hitchcock Medical Center, Lebanon, NH USA; 9 Clinical Pharmacology and Quantitative Pharmacology, Clinical Pharmacology and Safety Sciences, AstraZeneca, Gothenburg, Sweden; 10 Cardiovascular Safety Center of Excellence, CMO, Oncology R&D, AstraZeneca, Gothenburg, Sweden; 11 Integrated Bioanalysis, Clinical Pharmacology and Quantitative Pharmacology, BioPharmaceuticals R&D, AstraZeneca, Boston, MA USA; 12 Clinical Pharmacology and Quantitative Pharmacology, R&D Clinical Pharmacology and Safety Sciences Clinical Pharmacology, Waltham, MA USA

**Keywords:** Adavosertib, AZD1775, QT interval, Wee1 inhibitor, Pharmacokinetics, Pharmacodynamics

## Abstract

**Purpose:**

Adavosertib is a small-molecule, ATP-competitive inhibitor of Wee1 kinase. Molecularly targeted oncology agents have the potential to increase the risk of cardiovascular events, including prolongation of QT interval and associated cardiac arrhythmias. This study investigated the effect of adavosertib on the QTc interval in patients with advanced solid tumors.

**Methods:**

Eligible patients were ≥ 18 years of age with advanced solid tumors for which no standard therapy existed. Patients received adavosertib 225 mg twice daily on days 1–2 at 12-h intervals and once on day 3. Patients underwent digital 12-lead electrocardiogram and pharmacokinetic assessments pre-administration and time-matched assessments during the drug administration period. The relationship between maximum plasma drug concentration (C_max_) and baseline-adjusted corrected QT interval by Fridericia (QTcF) was estimated using a prespecified linear mixed-effects model.

**Results:**

Twenty-one patients received adavosertib. Concentration–QT modeling of ΔQTcF and the upper limit of the 90% confidence interval corresponding to the geometric mean of C_max_ observed on days 1 and 3 were below the threshold for regulatory concern (not > 10 ms). No significant relationship between ΔQTcF (vs baseline) and adavosertib concentration was identified (*P* = 0.27). Pharmacokinetics and the adverse event (AE) profile were consistent with previous studies at this dose. Eleven (52.4%) patients experienced 17 treatment-related AEs in total, including diarrhea and nausea (both reported in six [28.6%] patients), vomiting (reported in two [9.5%] patients), anemia, decreased appetite, and constipation (all reported in one [4.8%] patient).

**Conclusion:**

Adavosertib does not have a clinically important effect on QTc prolongation.

**ClinicalTrials.gov:**

NCT03333824.

**Supplementary Information:**

The online version contains supplementary material available at 10.1007/s00280-023-04555-2.

## Introduction

Wee1 is a protein kinase involved in the regulation of the cell cycle; inhibition of Wee1 leads to uncontrolled replication in the S phase and prevention of G2 cell-cycle arrest [1–6]. Adavosertib (AZD1775) is an orally active, first-in-class, small-molecule, ATP-competitive inhibitor of Wee1 kinase. Adavosertib treatment induces replication stress and endogenous DNA damage in tumor cells, which typically have defective DNA damage response pathways, resulting in mitotic catastrophe and cell death [3–6]. Adavosertib has been evaluated as mono- and combination therapy with chemotherapy (e.g. gemcitabine), olaparib, and durvalumab immunotherapy in numerous phase I and II studies in patients with a wide spectrum of solid tumors [7–12].

Patients receiving molecularly targeted therapeutic oncology agents that interfere with important cellular pathways, including survival and growth (e.g. multitargeted tyrosine kinase inhibitors, protein kinase C inhibitors, and vascular disruption agents), may be at increased risk of cardiovascular events, such as QT prolongation, hypertension, and congestive heart failure [13–15]. The duration of ventricular depolarization and subsequent repolarization is represented in the surface electrocardiogram (ECG) as the QT interval; a delay in cardiac repolarization – identifiable as prolongation of the QT interval – increases the risk of cardiac arrhythmias, including torsades de pointes (TdP) and other potentially lethal ventricular tachyarrhythmias [16]. Owing to its inverse relationship with heart rate (HR), the measured QT interval is routinely corrected to a value known as the QTc interval (QT interval corrected for HR); Bazett’s and Fridericia’s corrections are the most widely used [16].

Preclinical in vitro studies indicate that adavosertib is metabolized by, and is both a substrate for and an inhibitor of, cytochrome P450 enzymes [17]. A phase I study of adavosertib monotherapy in patients with advanced solid tumors determined the maximum tolerated dose to be 225 mg twice daily (bid) over 2.5 days per week per 21-day cycle [1]. Preclinical in vitro data indicate that adavosertib inhibits hERG potassium ion channels, and, in preliminary data from ongoing clinical studies, adverse drug reactions to adavosertib monotherapy include QTc prolongation (AstraZeneca, data on file, 2022).

The International Conference on Harmonisation (ICH) E14 guidelines for the evaluation of the effects of drugs on QT/QTc interval within the design of clinical studies recommend a “thorough QT/QTc analysis” in a dedicated study in healthy volunteers [16]. However, ICH guidance acknowledges that some drugs are not suitable for study in healthy volunteers because of issues related to safety and tolerability. In these cases, alternative approaches to a thorough QT/QTc analysis that take into account safety and ethical issues are recommended [16, 18].

Owing to the limited available data on the effect of adavosertib on ECG intervals, including the QTc interval, this study (NCT03333824) investigated the effect of adavosertib on the QTcF interval in patients most likely to benefit from adavosertib: those with advanced solid tumors.

## Methods

### Objectives

The primary objective of this study was to assess the effect of adavosertib on QTc following multiple oral doses in patients with advanced solid tumors. The primary QTc analysis parameter studied was the corrected QT interval by Fridericia (QTcF). The secondary objectives were to assess the adavosertib pharmacokinetic (PK)–pharmacodynamic (PD) relationship, specifically relating to digital ECG (dECG) parameters (HR [19], RR interval, PR interval, QRS interval), and to assess the safety and tolerability of adavosertib following single and multiple doses.

### Study design

**Fig. 1:**
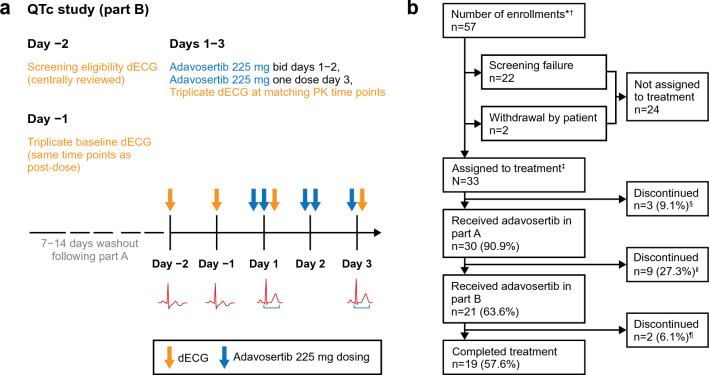
**a** Study design and **b** patient disposition flow chart. This manuscript focuses on pharmacodynamic data from part B of NCT03333824; part A is an adavosertib drug–drug interaction PK study, the results of which are reported separately. Refer to Fig. 1 for an overview of the study design of part B of the study. *Informed consent received; ^†^A number of patients enrolled more than once; there were 49 unique enrollments; ^‡^Study treatment refers to treatment with either ‘cocktail’ (of caffeine, omeprazole, and midazolam) or adavosertib; ^§^One each as a result of death (pancreatic cancer), study termination by the sponsor, and withdrawal by the patient; ^‖^Three each as a result of protocol-specific withdrawal and study termination by the sponsor, two because of adverse events (one was a case of diarrhea that was considered related to adavosertib), and one because of withdrawal by the patient; ^¶^Two were excluded from the PK analysis in part B because of gastrectomy and small-bowel resection. *bid* twice daily, *dECG* digital electrocardiogram, *PK* pharmacokinetic, *QTc* corrected QT interval

This manuscript focuses on the QTcF data from NCT03333824 (part B), a prospective, open-label, sequential (Fig. 1), non-randomized study conducted at seven clinical sites in the USA. Part A of NCT03333824 was an adavosertib drug–drug interaction PK study, the results of which are reported in a separate manuscript. Patients were screened for eligibility 28 days before the first adavosertib dose in part A; upon completion of part A, a washout period followed of at least 7, but no more than 14, days between the last dose in part A and day − 1 of this QTc study. On day − 2, patients underwent dECG to determine eligibility by central review. On day − 1, baseline dECG assessments were performed at clock times matched to the planned/scheduled dECG assessment times on days 1 and 3. Patients received adavosertib 225 mg bid on days 1–2 and once on day 3 (five doses; maximum tolerated/recommended phase II dose for combination therapy) at 12-h intervals. Doses were administered under fasting conditions (2 h pre-dose to 2 h post-dose), with prescribed anti-emetic(s) given orally 30 min prior to each dose [20]. On the morning of day 1, patients underwent dECG and PK assessments pre-dose (after anti-emetic administration) and for 12 h after receiving the adavosertib dose; each patient then received 3 × 75 mg adavosertib capsules (225 mg) bid for 2.5 days with 240 mL of water, administered under fasting conditions (2 h pre-dose to 2 h post-dose). On day 3, patients received their final adavosertib dose (30 min after anti-emetic administration) and underwent dECG and PK assessments pre-dose (after anti-emetic administration) and for a period of 12 h post-dose. On day 4, patients had their final (24-h) dECG/PK assessments relative to the adavosertib dose administered on day 3. Venous blood samples for study of adavosertib PK were taken on day 1 during multiple-dose administration and on day 3 following single-dose administration (Fig. 1). Upon completion of the study (i.e. collection of the 24-h dECG/PK sample and safety assessments on day 4), patients entered a 4-­day washout period relative to the last dose of adavosertib. Within 3 days after the washout period, patients attended an end-of-treatment visit, at which they were evaluated for their eligibility and interest to enroll in the open-label continued-access to adavosertib (NCT03313557).

Treatment with 1 mg of granisetron, an anti-emetic, orally 30 min prior to all adavosertib administrations was mandated; patients could also receive dexamethasone 4 mg orally or intravenously prior to each dose of adavosertib along with granisetron if medically indicated, and as long as consistent pretreatment conditions were maintained in the mornings prior to all serial ECG recordings, including on day − 1. This study was performed in accordance with the Declaration of Helsinki, Good Clinical Practice, applicable local regulatory requirements, and the AstraZeneca policy on bioethics [21]. All patients gave written informed consent prior to any study-defined procedures being performed.

### Patients

Patients were eligible for study inclusion if they met the following criteria: ≥ 18 years of age; histologically or cytologically confirmed locally advanced or metastatic solid tumor, excluding lymphoma, for which standard therapy did not exist or had proven ineffective or intolerable; any prior palliative radiation had to be completed at least 7 days prior to the start of study treatment, and patients had to have recovered from any acute adverse effects prior to the start of study treatment; assessed as Eastern Cooperative Oncology Group performance status of 0 or 1; no abnormalities in baseline laboratory values within 7 days of study treatment initiation.

Key exclusion criteria included: QTcF interval > 450 ms or congenital long-QT syndrome; clinically significant cardiovascular symptoms at the time of screening or within the previous 6 months.

### Electrocardiogram assessments

Digital ECGs were recorded in triplicate to determine absolute values and time-matched changes from baseline for the ECG intervals. dECGs were conducted on days − 1, 1, and 3, before and at 0.5, 1, 2, 3, 4, 6, 8, 12, and 24 h after receiving the adavosertib dose. Patients had their final dECG/PK assessments on day 4, 24 h post-dose. Baseline for dECG assessments was defined as the time-matched observation on day − 1. Triplicate dECG recordings were taken within an approximate 5-min period prior to PK sample collection and as close to the scheduled times as possible. Patients rested for ≥ 10 min prior to dECG recording and were in the same supine body position for all recordings. HR and RR, QT, PR, and QRS interval parameters were recorded. Standardized equipment was provided by the central ECG laboratory. Recorded dECGs were sent to the central ECG laboratory for review and interpretation, using their standard operating procedures and with manual adjudication (3 beat on lead II).

### Pharmacokinetic assessment

Venous blood PK samples were taken on day 1 before dosing and 0.25, 0.5, 0.75, 1, 2, 3, 4, 6, 8, and 12 h after adavosertib dosing, and on day 3 before dosing and 0.25, 0.5, 0.75, 1, 2, 3, 4, 6, 8, 12, and 24 h after adavosertib dosing. Patients had their final PK assessments on day 4, 24 h post-dose. Recorded PK parameters included: area under the plasma concentration–time curve from 0 to 12 h (AUC_0–12_); maximum plasma drug concentration (C_max_); time to reach maximum plasma drug concentration (t_max_); minimum plasma drug concentration (C_min_; day 3 only); geometric least-squares (LS) mean ratios for AUC_0–12_ and C_max_; fluctuation index over a dosing interval; and apparent clearance at steady state. The concentration of adavosertib in human plasma was determined by Labcorp Bioanalytical Laboratories (Madison, WI, USA) using the bioanalytical method described previously [22]. The method was initially developed and validated by Merck & Co and was transferred and cross-validated by Labcorp Bioanalytical Laboratories; the assay had a linear dynamic range of 2–1000 ng/mL, with a lower limit of quantification of 2 ng/mL [23]. The precision (percentage coefficient of variation) was ≤ 4.2%, and the accuracy (percentage bias) of the quality control samples was within 0.7–1.5%.

Reproducibility was confirmed by re-analysis of a random selection of 87 samples with adavosertib concentrations above the lower limit of quantification; 98.9% of the results obtained following the initial and repeat analysis were within 20.0% of the mean of the two values, thus meeting the acceptance criteria [24]. All samples were stored at − 70 °C prior to analysis and analyzed within the validated time frame.

### Safety and tolerability assessments

Safety was appraised throughout the study by the assessment of adverse events (AEs; graded by Common Terminology Criteria for Adverse Events [CTCAE], version 4.03), physical examination, and evaluation of vital signs, ECG, and laboratory data (clinical chemistry and hematology). Dose reductions were not allowed.

### Concentration–QTc model

A plasma-concentration-to-QTc relationship analysis (described previously by Garnett et al. [19]) was conducted using a prespecified linear mixed-effects model in which the ‘baseline-corrected’ (day − 1 corrected) change from baseline in QTcF was the dependent variable and the adavosertib plasma concentration obtained on days 1 and 3 was the independent variable. Estimates were made of the mean change in QTcF at the geometric mean C_max_ on days 1 and 3 from the fitted model, with corresponding 90% two-sided confidence intervals (CIs).

### Statistical analyses

No formal sample size estimation was conducted for this study. The number of patients was based on careful clinical consideration to gain adequate information on the primary endpoints while exposing as few patients as possible to study procedures. Enrollment of approximately 30 patients, with a target of 20 evaluable patients, was considered adequate and sufficient to meet the objectives of this study.

The safety analysis set included all patients who received ≥ 1 dose of adavosertib. A treatment-emergent AE was defined as an AE with the start date and time on or after the first dose of adavosertib on day 1 up to and including the end-of-study visit (for patients who did not enroll in the open-label continued-access study) or end-of-treatment visit (for patients who enrolled in the open-label continued-access study), or, for pre-existing pretreatment AEs, the date on which they worsened in severity after the first dose of study treatment.

The PD analysis set included all dosed patients who had ≥ 1 evaluable time-matched PD endpoint (QTcF on day − 1 and day 1 or 3) after first administration of adavosertib without protocol deviations or events that would affect the PD analysis, and the PK analysis set included all dosed patients who had ≥ 1 quantifiable plasma concentration for adavosertib collected post-dose without protocol deviations or events that would affect the PK analysis.

## Results

### Patients

Of 57 enrolled patients (from 49 unique patients, as several patients were enrolled more than once because of rescreening), 33 were assigned to treatment and 21 received adavosertib in this PD study (summarized in Fig. 1). Nineteen (90.5%) patients completed the study (two discontinued because of AEs). All 21 patients who received adavosertib in this study investigating the effects of adavosertib on the QTc interval were included in both the PD and safety analysis sets, while two patients were excluded from the PK analysis because they had undergone gastrectomy and small-bowel resection (Fig. 1). Patient baseline characteristics are summarized in Table 1. Gender distribution was balanced, median weight was 75.8 kg (range 46–103), median age was 61 years (range 41–74), and median body mass index was 28.0 kg/m^2^ (range 17.5–36.9).

**Table 1:** Patient demographics and disease characteristics (safety analysis set)

Characteristic	Safety analysis set (*n* = 21)
Age, years	
Mean (SD)	59.3 (8.7)
Median (range)	61 (41–74)
Age group, *n* (%)	
40 to < 50 years	4 (19.0)
50 to < 65 years	12 (57.1)
≥ 65 years	5 (23.8)
Sex, *n* (%)	
Male	9 (42.9)
Female	12 (57.1)
Race, *n* (%)	
Asian	1 (4.8)
Black or African American	3 (14.3)
White	17 (81.0)
Body mass index, kg/m^2^	
* n*	19
Mean (SD)	27.0 (5.4)
Median (range)	28.0 (17.5–36.9)
ECOG performance status, *n* (%)	
0 (normal activity)	11 (52.4)
1 (restricted activity)	10 (47.6)
AJCC stage at primary diagnosis, *n* (%)^a^	
IA	3 (14.3)
IB	0
IIA	2 (9.5)
IIB	2 (9.5)
IIIA	2 (9.5)
IIIB	1 (4.8)
IV	11 (52.4)
Primary tumor location, *n* (%)	
Pancreas	3 (14.3)
Colon	3 (14.3)
Lung	2 (9.5)
Ovary	3 (14.3)
Peritoneum	2 (9.5)
Other^b^	8 (38.1)

ECOG performance status and overall disease classification are based on assessments at baseline. AJCC staging, primary tumor location, tumor grade, and histology type are based on assessments at primary diagnosis

*AJCC* American Joint Committee on Cancer, *ECOG* Eastern Cooperative Oncology Group, *SD* standard deviation

^a^All patients enrolled in this study had disease progression to unresectable recurrent/metastatic cancer since diagnosis

^b^Other includes appendix, breast, gastric cardia, head and neck, larynx, prostate gland, rectum, and uterus (all *n* = 1)

Two patients used disallowed concomitant medication: one patient took famotidine from day 30 for 11 days because of an AE of dyspepsia – this deviation did not impact the primary endpoint of the study; the other patient took fentanyl for tumor-related pain on days 39, 45, and 60 of the study – this did not affect the primary or secondary endpoints of the study. Important protocol deviations included: one patient had day 1 ECG data excluded due to receipt of dexamethasone in addition to granisetron on day 1, but not on day − 1 and day 3; one patient had day 3, 12-h ECG data excluded as the adavosertib dose was taken during a vomiting episode; and one patient had their day 3 pre-dose ECG excluded because there was no paired pre-dose adavosertib PK sample. ECG replicates were excluded when found to be taken after the paired PK blood collection or because the ECG evaluation was not interpretable. All patients with ECG replicates taken after the PK blood collection had sufficient appropriately paired concentration–ECG data for inclusion in the PD analysis set.

### Effect of adavosertib on QTcF

Concentration–QTc model predictions of ΔQTcF at the geometric mean adavosertib C_max_ were − 2.4 ms (90% CI − 5.9, 1.1) and − 0.8 ms (90% CI − 5.1, 3.6) on day 1 (712.8 nM) and day 3 (1462 nM), respectively (Table 2).

**Table 2:** Concentration–QT model predictions of ΔQTcF at the geometric mean of adavosertib C_max_

Day	Geometric mean C_max_, nM	Mean change in QTcF, ms	90% CI
Day 1	712.8	− 2.4	− 5.9, 1.1
Day 3	1462.0	− 0.8	− 5.1, 3.6

*CI* confidence interval, *C*_*max*_ maximum plasma drug concentration, *QTcF* corrected QT interval by Fridericia

**Fig. 2:**
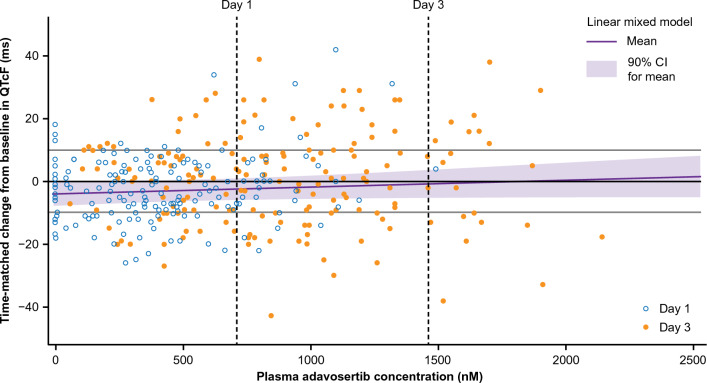
Time-matched change in QTcF from baseline versus adavosertib concentration (PD analysis set). Regression line and 90% confidence bands for ΔQTcF from baseline were estimated using a linear mixed-effects model. Dashed vertical lines indicate the geometric mean C_max_ of adavosertib on days 1 and 3. *CI* confidence interval, *PD* pharmacodynamic, *QTcF* corrected QT interval by Fridericia

Time-matched ΔQTcF versus adavosertib concentration is presented in Fig. 2 (days 1 and 3 combined); no significant relationship between ΔQTcF (vs baseline) and adavosertib concentration was identified (*P* = 0.27; Supplementary Table S1). There was no apparent hysteresis and a linear fit was appropriate for the data.

Following a single oral dose of adavosertib 225 mg on day 1, the largest mean difference in ΔQTcF from baseline was 1.1 ms (mean [min, max] 401.1 [341.0, 422.0]), observed 3 h post-dose; following multiple dosing (five doses) of adavosertib 225 mg (day 3), the largest mean difference in ΔQTcF from baseline was 7.3 ms (mean [min, max] 405.7 [381.0, 431.0]), 3 h post-dose (median adavosertib t_max_ was 3.0 h). Observed QT interval, QTcF values, and time-matched mean changes from baseline are shown in Supplementary Table S2.

### Effect of adavosertib on dECG parameters

On days 1 and 3, there were no apparent effects of adavosertib on HR; mean change in HR ranged from − 1.7 to 2.9 beats per minute (bpm) on day 1 and from 1.4 to 9.5 bpm on day 3. The mean change was within ± 10 bpm and thus not considered clinically significant or to affect the performance of the correction of QT for HR using the Fridericia formula (Supplementary Table S3).

There were no apparent effects of adavosertib on PR interval; mean change in PR interval ranged from − 4.4 to 3.5 ms on day 1 and from − 4.4 to 0.3 ms on day 3 (Supplementary Table S3). There were no apparent effects of adavosertib on QRS interval; mean change in QRS interval ranged from − 1.2 to 1.4 ms on day 1 and from − 1.8 to − 2.0 ms on day 3 (Supplementary Table S3). There were also no apparent effects of adavosertib on RR interval; mean change in RR interval ranged from − 38.0 to 22.3 ms on day 1 and from − 102.5 to − 30.4 ms on day 3 (Supplementary Table S3).

### Adequacy of QT correction (QTc) formulae

**Fig. 3:**
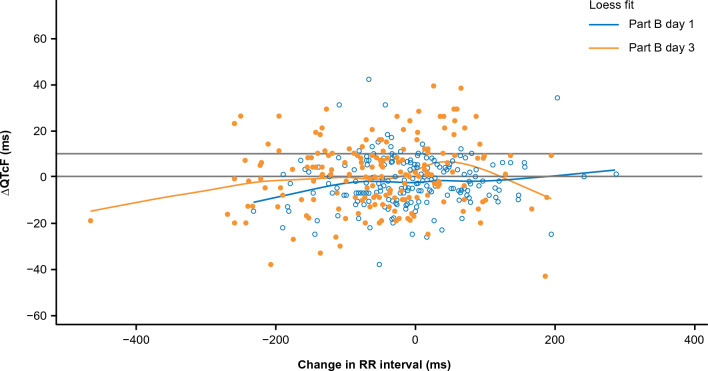
Individual time-matched change in QTcF from baseline over time versus change in RR interval (safety analysis set). *LOESS* locally estimated scatter-plot smoothing, *QTcF* corrected QT interval by Fridericia

Model diagnostics indicated that ΔQTcF did not appear to be correlated with changes in RR interval, suggesting that the QT interval is independent of HR and that QTcF is an adequate correction factor (Fig. 3). Model residual diagnostics demonstrated that model covariates were adequate, assumption of normality was met, and a linear fit was suitable for the data. Observed versus predicted ΔQTcF values showed good overall concordance (Supplementary Figure S1).

### Adavosertib pharmacokinetic profile

Geometric mean AUC_0–12_ and C_max_ of adavosertib on day 1 were 4940.0 nM·h and 712.8 nM, respectively, versus 10,810.0 nM·h and 1462.0 nM on day 3. Median t_max_ of adavosertib was 3.0 h (range 1.0–6.0) on day 1 and 2.5 h (range 1.0–3.1) on day 3, and C_min_ on day 3 was 410.4 nM (Supplementary Table S4). A summary of the AUC_0–12_ and C_max_ geometric LS mean ratios and CIs following once daily (qd) dosing (day 3) and bid dosing (day 1) to assess accumulation is shown in Supplementary Table S5. Accumulation over 2.5 days of bid dosing was approximately 2.3-fold and 2.1-fold for AUC_0–12_ and C_max_, respectively (Supplementary Table S5 and Supplementary Figure S2). The adavosertib concentration in human plasma ranged from 4 to 2000 nM.

### Safety

No patient had QTcF > 450 ms or an increase in QTcF of > 60 ms over the treatment period (days 1–3, 24 h post-dose). On day 1, two patients had an increase in QTcF of > 30 ms (Table 3). One patient took the antidepressant mirtazapine, there being a positive relationship between its concentration and prolongation of QTc interval, and had increases in QTcF of > 30 ms only on day 3, pre-dose and 4 h post-dose; measurements at all other time points were within normal limits (Table 3) [25]. One patient received sertraline (associated with a conditional risk of TdP). Neither sertraline nor mirtazapine were prohibited; therefore, ECG data of the patients who received these drugs were included in the primary ECG analyses.

**Table 3:** QTcF outliers at any observation on treatment (PD analysis set)

	Patients, *n* (%)	
	Day 1 (*N* = 21)	Day 3 (*N* = 20)
Value above threshold at any time during treatment		
> 450 ms^a^	0	0
> 480 ms^a^	0	0
> 500 ms^a^	0	0
Increase more than threshold at any time during treatment^b^		
> 30 ms^a^	2 (9.5)	1 (4.8)
> 60 ms^a^	0	0
Value above threshold for both absolute value and change from baseline at any time during treatment^a^		
Value > 450 ms and increase > 30 ms^a^	0	0
Value > 500 ms and increase > 60 ms^a^	0	0

*PD* pharmacodynamic, *QTcF* corrected QT interval by Fridericia

^a^The number of subjects is a cumulative count for each category. Baseline is the time-matched observation on day 1

^b^Change from baseline to any observation on treatment. ‘On treatment’ is defined as between the start of treatment and 30 days following the date of last dose of study medication

AEs were recorded for 16/21 (76.2%) patients who received adavosertib; the most common AEs were gastrointestinal events such as diarrhea, nausea (each in seven [33.3%] patients), and vomiting (five [23.8%] patients). Eleven (52.4%) patients experienced a total of 17 treatment-related AEs (diarrhea and nausea [both reported in six (28.6%) patients], vomiting [two (9.5%) patients], anemia, decreased appetite, and constipation [all reported in one (4.8%) patient]); four events were CTCAE grade 3 (anemia, hypotension, diarrhea, and nausea [all reported in one (4.8%) patient]), with the remainder grade 1 or 2 (Supplementary Table S6). One (4.8%) patient had a serious AE (vomiting) that was not considered drug related. No AEs with an outcome of death were observed. Two (9.5%) patients discontinued treatment because of AEs: one (4.8%) because of diarrhea, nausea, and vomiting; and one (4.8%) because of nausea and vomiting.

## Discussion

This study evaluated the effect of adavosertib on QTcF in patients with advanced solid tumors. No significant relationship was found between ΔQTcF and adavosertib concentration, and administration of adavosertib had no clinically relevant effect on recorded ECG parameters. The upper limits of the model-predicted 90% CIs for ΔQTcF were well below 10 ms, supporting the conclusion that adavosertib does not have a clinically relevant impact on cardiac repolarization, and the concentration–QTc model diagnostics support its adequacy.

Three patients received medication associated with either a conditional risk of TdP (sertraline) or a potential risk of TdP (mirtazapine). When a sensitivity analysis was performed that excluded these patients from the concentration–QTc modeling, the upper limits of the predicted 90% CIs for ΔQTcF were substantially lower (< 1.5 ms). This further supports the conclusion that adavosertib monotherapy at this dose and schedule does not have a clinically relevant impact on cardiac repolarization.

The results of the present study build on the evidence of the PK and PD properties of adavosertib gathered to date [22, 26]. Adavosertib accumulation (approximately twofold) over the dosing period was concordant with results from previous clinical studies at this dose [1]. The secondary PK objectives of this study included describing the PKs of adavosertib following single- and multiple-dose administration. Adavosertib exposure was consistent with that in previous studies at this dose; therefore, the adavosertib exposures observed in this study were representative of exposures expected in the clinical setting [1]. In a previous phase I study of adavosertib in patients with advanced refractory solid tumors (NCT01748825), adavosertib 200–400 mg qd was evaluated 5 days on/2 days off for 2 weeks of a 21-day schedule. Preliminary PK data from the qd dosing showed that exposure increased more than dose proportionally between 200 and 300 mg after both single and multiple doses [1]. In a recently completed phase Ib trial (NCT02482311) assessing the safety, tolerability, and efficacy of adavosertib as monotherapy in patients with advanced solid tumors, adavosertib accumulation (based on AUC_0–12_) was approximately 2.1 (8100 nM·h) after 200 mg bid dosing (21-day cycle; PK samples taken on cycle 1 day 3 or day 10, or cycle 2 day 10) [7]. In the present study, accumulation over 2.5 days of bid dosing was approximately 2.3: 4791 nM·h (AUC_0–12_) on day 1, after 1 day of 225 mg bid dosing, and 11,060 nM·h after 225 mg qd dosing on day 3.

This study was not designed (no placebo or positive control) or powered for hypothesis testing in a by-time-point analysis of the QTcF parameter. As indicated in the ICH E14 (R3) questions and answers, a by-time-point analysis (intersection–union test or point estimate and CIs) is not sufficient for making inferences regarding QTc prolongation when a study is not powered to assess the magnitude of QT prolongation for each time point [16].

Compared with the recommended phase II dose for adavosertib monotherapy (300 mg qd, 5 days on/2 days off for 2 of 3 weeks), the adavosertib dose in this study (225 mg bid) was higher and the dosing duration (2.5 days) was sufficient to reach steady state. Therefore, the effect of adavosertib on ECG intervals, including the QTc interval, at the recommended phase II dose is not expected to be greater than that observed during this study [27]. Drugs that prolong the QTc interval generally contain warnings in their labels to address the associated safety concerns; in terms of further clinical development, it is therefore reassuring that adavosertib is not associated with a significant effect on the QTc interval [28].

The most common treatment-related AEs were of CTCAE grade 1–2 and related to the gastrointestinal system (diarrhea, nausea, and vomiting), despite administration of anti-emetic prophylaxis; nausea and vomiting are part of the known toxicity profile of adavosertib. The safety profile observed in this study was consistent with that in previous studies of adavosertib administered as monotherapy [1, 7, 20].

## Conclusions

Concentration–QTc modeling of time-matched PK and ECG samples from this study was used to assess the risk of QTc prolongation with adavosertib. In patients with advanced solid tumors who received adavosertib 225 mg bid, ΔQTcF and the upper limit of the 90% CI corresponding to the C_max_ on days 1 and 3 were well below 10 ms. It can therefore be concluded that adavosertib does not prolong the QTcF interval at clinically relevant drug exposures.

## Supplementary Information

Below is the link to the electronic supplementary material.

Supplementary file1 (PDF 362 KB)

## Data Availability

Data underlying the findings described in this manuscript may be obtained in accordance with AstraZeneca’s data sharing policy described at https://astrazenecagrouptrials.pharmacm.com/ST/Submission/Disclosure. Data for studies directly listed on Vivli can be requested through Vivli at www.vivli.org. Data for studies not listed on Vivli could be requested through Vivli at https://vivli.org/members/enquiries-about-studies-not-listed-on-the-vivli-platform/. AstraZeneca Vivli member page is also available outlining further details: https://vivli.org/ourmember/astrazeneca/.
