# Supplementary material for: Adavosertib (AZD1775) does not prolong the QTc interval in patients with advanced solid tumors: a phase I open-label study
Source: Cancer Chemother Pharmacol. 2023 Jun 27;92(2):141–50. doi: 10.1007/s00280-023-04555-2 (PMC10326086; doi:10.1007/s00280-023-04555-2)
Supplement: Supplementary file 1 — Supplementary file1 (PDF 362 KB) [file 280_2023_4555_MOESM1_ESM.pdf]

Supplementary material

Adavosertib (AZD1775) does not prolong the QTc interval in patients with advanced solid tumors: a phase I open-label study

Någård M, Ah-See M-L, Strauss J, et al.

Supplementary Fig. S1 Residual diagnostics for analysis of time-matched change in QTcF from baseline versus plasma adavosertib concentration: observed versus individual predicted (pharmacodynamic analysis set)

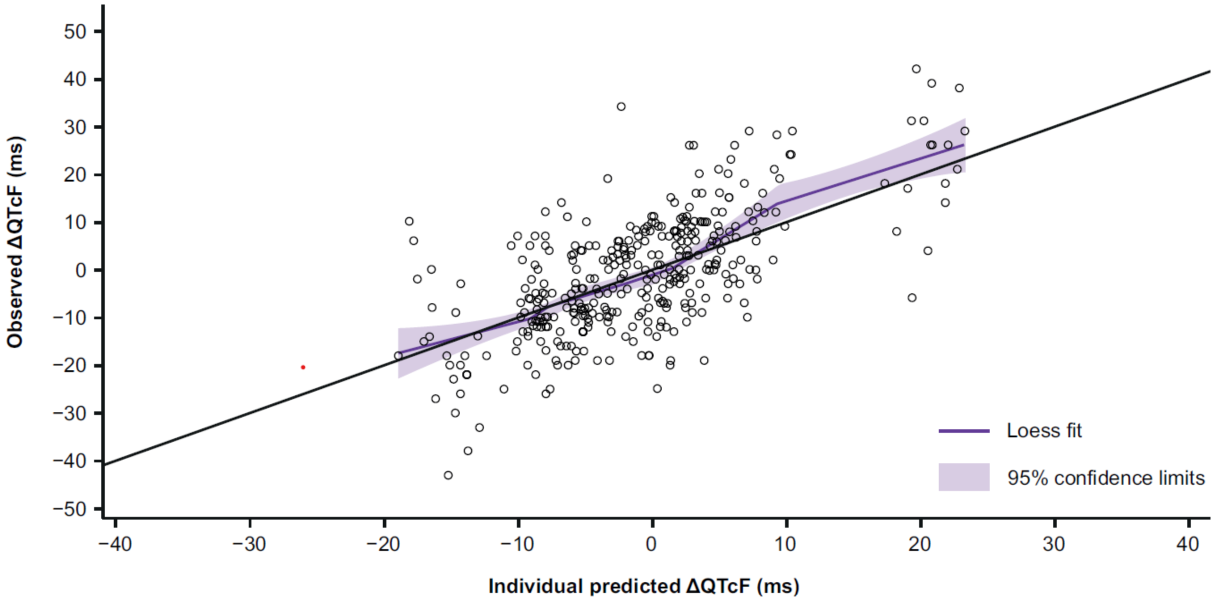

LOESS locally estimated scatter-plot smoothing, *QTcF* corrected QT interval by Fridericia

Supplementary Fig. S2 Individual and geometric mean $AUC_{0–12}$ and C_{max} of adavosertib after single (day 3) and multiple (day 1) doses (PK analysis set)

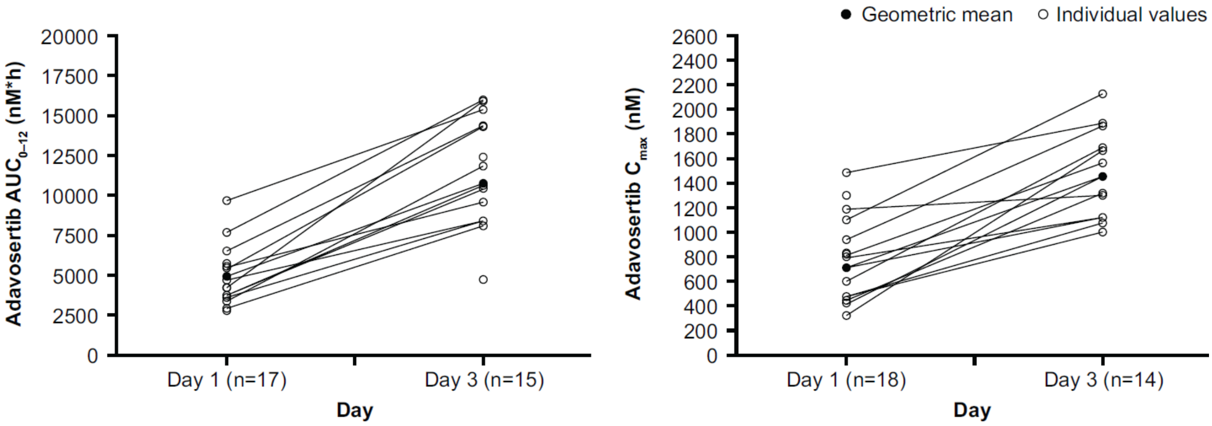

Treatment: adavosertib 225 mg (3 x 75 mg capsules) bid on day 1, qd on day 3

$AUC_{0–12}$ area under the plasma concentration–time curve from 0 to 12 hours, *bid* twice daily, *C_{max}* maximum plasma drug concentration, *PK* pharmacokinetic, *qd* once daily

Supplementary Table S1. Statistical assessment of the relationship between adavosertib concentration and time-matched ΔQTcF

Model effect	Estimate	95% CI	*P* value
Intercept	−2.4	−6.2, 1.4	0.21
Concentration (linear model)[a]	0.005	−0.004, 0.01	0.27
Day	−1.6	−4.3, 1.0	0.23

[a]Slope

CI confidence interval, *QTcF* corrected QT interval by Fridericia

Supplementary Table S2. Observed ECG variables (QT interval and QTcF) and time-matched change from baseline for days 1 and 3 following adavosertib dosing (safety analysis set, *N* = 21)

	QT interval, ms<br>(*n*)<br>Mean (change from baseline)<br>(minimum, maximum)		QTcF, ms<br>(*n*)<br>Mean (change from baseline)<br>(minimum, maximum)	
	**Day 1**	**Day 3**	**Day 1**	**Day 3**
Pre-dose	(19)<br>373.2 (−3.0)<br>(331.0, 427.0)	(18)<br>369.8 (−8.0)<br>(317.0, 430.0)	(19)<br>395.4 (−0.3)<br>(357.0, 426.0)	(18)<br>397.9 (−0.6)<br>(361.0, 433.0)
0.5 hours	(18)<br>383.3 (−3.9)<br>(328.0, 416.0)	(16)<br>383.6 (−4.9)<br>(347.0, 430.0)	(18)<br>397.5 (−6.3)<br>(377.0, 420.0)	(16)<br>399.9 (−0.4)<br>(369.0, 426.0)
1 hour	(20)<br>386.1 (+0.7)<br>(341.0, 439.0)	(18)<br>381.6 (−3.9)<br>(334.0, 429.0)	(20)<br>397.8 (−2.4)<br>(354.0, 417.0)	(18)<br>398.4 (−0.2)<br>(357.0, 425.0)
2 hours	(20)<br>383.4 (−1.3)<br>(328.0, 424.0)	(16)<br>382.3 (−3.8)<br>(347.0, 438.0)	(20)<br>399.1 (−2.1)<br>(349.0, 435.0)	(16)<br>402.7 (+2.9)<br>(366.0, 430.0)
3 hours	(21)<br>380.0 (−4.5)<br>(335.0, 416.0)	(17)<br>381.5 (−3.3)<br>(345.0, 416.0)	(21)<br>401.1 (+1.1)<br>(341.0, 422.0)	(17)<br>405.7 (+7.3)<br>(381.0, 431.0)

4 hours	(21)	(19)	(21)	(19)
	377.2 (−3.0)	372.4 (−7.8)	400.4 (−0.7)	396.5 (−2.7)
	(339.0, 415.0)	(325.0, 403.0)	(358.0, 425.0)	(373.0, 438.0)
6 hours	(20)	(18)	(20)	(18)
	367.3 (−7.9)	366.8 (−9.0)	396.0 (−4.0)	397.2 (−1.2)
	(329.0, 398.0)	(323.0, 398.0)	(355.0, 427.0)	(372.0, 432.0)
8 hours	(20)	(19)	(20)	(19)
	369.0 (−5.1)	369.1 (−7.9)	395.2 (−3.9)	396.3 (−1.8)
	(327.0, 400.0)	(310.0, 403.0)	(359.0, 418.0)	(368.0, 436.0)
12 hours	(20)	(19)	(20)	(19)
	367.7 (+0.2)	364.9 (−6.1)	392.9 (−2.2)	395.8 (+1.8)
	(334.0, 397.0)	(313.0, 395.0)	(352.0, 419.0)	(359.0, 428.0)
24 hours	(21)	(17)	(21)	(17)
	367.7 (−6.9)	358.6 (−14.5)	392.2 (−4.2)	396.2 (+1.0)
	(295.0, 401.0)	(310.0, 398.0)	(352.0, 416.0)	(367.0, 435.0)
End-of-	(20)		(20)	
treatment	373.2		400.3	
follow-up	(325.0, 424.0)		(380.0, 426.0)	

ECG electrocardiogram, *QTcF* corrected QT interval by Fridericia

Supplementary Table S3. Observed ECG variables (heart rate, RR interval, PR interval, and QRS interval) and time-matched change from baseline for days 1 and 3 following adavosertib dosing (safety analysis set, *N* = 21)

	**Heart rate, bpm (*n*) Mean (change from baseline) (minimum, maximum)**		**RR interval, ms (*n*) Mean (change from baseline) (minimum, maximum)**		**PR interval, ms (*n*) Mean (change from baseline) (minimum, maximum)**		**QRS interval, ms (*n*) Mean (change from baseline) (minimum, maximum)**	
	**Day 1**	**Day 3**	**Day 1**	**Day 3**	**Day 1**	**Day 3**	**Day 1**	**Day 3**
Pre-dose	(19)<br>72.9 (+1.3)<br>(55.0, 107.0)	(18)<br>76.1 (+3.9)<br>(59.0, 109.0)	(19)<br>853.1 (−19.7)<br>(564.0, 1086.0)	(18)<br>815.7 (−47.4)<br>(548.0, 1023.0)	(19)<br>156.7 (−0.5)<br>(126.0, 198.0)	(18)<br>152.3 (−4.4)<br>(124.0, 199.0)	(19)<br>90.8 (−0.7)<br>(76.0, 125.0)	(18)<br>91.2 (+0.1)<br>(71.0, 123.0)
0.5 hours	(18)<br>68.2 (−1.3)<br>(53.0, 92.0)	(16)<br>68.9 (+1.8)<br>(58.0, 88.0)	(18)<br>907.1 (+16.0)<br>(651.0, 1132.0)	(16)<br>887.1 (−39.1)<br>(682.0, 1030.0)	(18)<br>161.6 (+2.3)<br>(131.0, 205.0)	(16)<br>159.1 (0.0)<br>(131.0, 195.0)	(18)<br>90.9 (−0.2)<br>(77.0, 128.0)	(16)<br>93.3 (+1.1)<br>(83.0, 130.0)
1 hours	(20)<br>67.1 (−1.7)<br>(51.0, 94.0)	(18)<br>69.4 (+1.4)<br>(56.0, 91.0)	(20)<br>926.5 (+22.3)<br>(639.0, 1167.0)	(18)<br>884.9 (−30.4)<br>(663.0, 1064.0)	(20)<br>161.9 (+3.2)<br>(132.0, 203.0)	(18)<br>157.2 (−0.4)<br>(124.0, 206.0)	(20)<br>91.2 (+1.4)<br>(77.0, 128.0)	(18)<br>92.4 (+2.0)<br>(79.0, 123.0)
2 hours	(20)<br>69.3 (−0.8)<br>(52.0, 100.0)	(16)<br>71.4 (+2.5)<br>(57.0, 90.0)	(20)<br>899.0 (+6.3)<br>(600.0, 1163.0)	(16)<br>859.1 (−53.0)<br>(664.0, 1058.0)	(20)<br>162.0 (+3.5)<br>(130.0, 202.0)	(16)<br>157.7 (+0.3)<br>(125.0, 205.0)	(20)<br>91.3 (+0.7)<br>(78.0, 131.0)	(16)<br>93.3 (+1.8)<br>(83.0, 122.0)
3 hours	(21)<br>71.8 (+2.9)<br>(53.0, 100.0)	(17)<br>72.9 (+4.8)<br>(53.0, 84.0)	(21)<br>860.9 (−38.0)<br>(600.0, 1125.0)	(17)<br>838.1 (−74.5)<br>(714.0, 1117.0)	(21)<br>158.5 (−1.9)<br>(127.0, 198.0)	(17)<br>157.2 (−1.4)<br>(134.0, 193.0)	(21)<br>91.5 (−0.6)<br>(76.0, 132.0)	(17)<br>94.1 (+1.1)<br>(85.0, 124.0)
4 hours	(21)<br>73.1 (+1.4)<br>(53.0, 109.0)	(19)<br>73.5 (+2.7)<br>(56.0, 92.0)	(21)<br>848.3 (−13.0)<br>(550.0, 1144.0)	(19)<br>836.0 (−37.2)<br>(656.0, 1069.0)	(21)<br>158.6 (+0.1)<br>(131.0, 195.0)	(19)<br>156.9 (−0.3)<br>(127.0, 186.0)	(21)<br>91.7 (−0.5)<br>(76.0, 131.0)	(19)<br>92.5 (0.0)<br>(79.0, 128.0)

6 hours	(20)<br>76.2 (+2.4)<br>(54.0, 98.0)	(18)<br>77.3 (+4.6)<br>(55.0, 96.0)	(20)<br>809.8 (−25.5)<br>(614.0, 1116.0)	(18)<br>794.3 (−54.7)<br>(629.0, 1100.0)	(20)<br>155.1 (−4.4)<br>(129.0, 189.0)	(18)<br>154.7 (−3.6)<br>(126.0, 191.0)	(20)<br>91.6 (+0.3)<br>(78.0, 135.0)	(18)<br>92.6 (+0.6)<br>(77.0, 127.0)
8 hours	(20)<br>75.1 (+1.0)<br>(56.0, 101.0)	(19)<br>75.4 (+3.3)<br>(57.0, 102.0)	(20)<br>823.5 (−11.2)<br>(593.0, 1079.0)	(19)<br>816.2 (−43.9)<br>(591.0, 1062.0)	(20)<br>154.5 (−2.5)<br>(128.0, 194.0)	(19)<br>157.4 (−0.1)<br>(124.0, 196.0)	(20)<br>90.5 (−1.2)<br>(78.0, 129.0)	(19)<br>90.7 (−1.8)<br>(75.0, 126.0)
12 hours	(20)<br>74.2 (−1.5)<br>(58.0, 92.0)	(19)<br>77.7 (+4.7)<br>(57.0, 102.0)	(20)<br>828.0 (+13.0)<br>(655.0, 1023.0)	(19)<br>792.9 (−51.5)<br>(588.0, 1052.0)	(20)<br>154.7 (−1.5)<br>(130.0, 191.0)	(19)<br>154.5 (−2.8)<br>(123.0, 195.0)	(20)<br>91.4 (+0.1)<br>(80.0, 124.0)	(19)<br>90.9 (−1.1)<br>(81.0, 122.0)
24 hours	(21)<br>74.7 (+2.1)<br>(54.0, 117.0)	(17)<br>82.7 (+9.5)<br>(64.0, 110.0)	(21)<br>832.5 (−23.1)<br>(513.0, 1103.0)	(17)<br>747.9 (−102.5)<br>(550.0, 941.0)	(21)<br>156.9 (−1.1)<br>(132.0, 198.0)	(17)<br>152.3 (−3.4)<br>(127.0,197.0)	(21)<br>91.2 (+0.5)<br>(77.0, 133.0)	(17)<br>91.8 (+1.2)<br>(81.0, 136.0)
End-of-treatment follow-up	(20)<br>76.0<br>(51.0, 111.0)		(20)<br>819.3<br>(544.0, 1170.0)		(20)<br>155.5<br>(129.0, 192.0)		(20)<br>91.8<br>(77.0,132.0)	

ECG electrocardiogram

Supplementary Table S4. Summary of PK parameters of adavosertib for days 1 and 3 (PK analysis set)

	**$AUC_{0–12}$, nM·h**	**C_{max}, nM**	**C_{min}, nM**	**C_{avg}, nM**	**t_{max}, h**	**CL_{ss}/F, L/h**	**FI, %**	**$RAUC_{0–12}$**	**RC_{max}**
**Day 1 (*N* = 19)**									
n	17	18	ND	ND	18	ND	ND	ND	ND
Geometric mean	4940.0	712.8	ND	ND	ND	ND	ND	ND	ND
CV, %	39.5	45.0	ND	ND	ND	ND	ND	ND	ND
Median (minimum, maximum)	5383.0 (2730.0, 9650.0)	759.0 (326.0, 1490.0)	ND	ND	3.0 (1.0, 6.0)	ND	ND	ND	ND
**Day 3 (*N* = 17)**									
n	15	14	15	15	14	15	15	13	13
Geometric mean	10,810.0	1462.0	410.4	901.2	ND	ND	ND	2.4	2.1
CV, %	34.15	24.0	136.1	34.3	ND	ND	ND	28.5	44.3
Arithmetic mean (SD)	11,340.0 (3356.0)	1500.0 (348.3)	517.7 (223.4)	944.8 (279.4)	ND	43.2 (16.8)	117.0 (63.4)	2.5 (0.7)	2.3 (1.1)
Median (minimum, maximum)	10,630.0 (4710.0, 16,000.0)	1515.0 (1010.0, 2140.0)	499.0 (12.0, 931.0)	898.9 (390.0, 1330.0)	2.5 (1.0, 3.1)	41.5 (27.7, 93.7)	102.4 (58.2, 332.7)	2.3 (1.6, 3.8)	2.0 (1.1, 5.1)

Treatment: adavosertib 225 mg (3 x 75 mg capsules) bid on days 1–2 and qd on day 3. CV calculated as $100 \times \sqrt{[\exp(s^2) - 1]}$, where s is the SD of the data on a log scale

$AUC_{0–12}$ area under the plasma concentration–time curve from 0 to 12 hours, *C_{avg}* average concentration over a dosing interval, *CL_{ss}/F* clearance at steady state, *C_{min}* minimum plasma drug concentration, *CV* geometric coefficient of variation, *FI* fluctuation index over a dosing interval, *ND* not determined, *PK* pharmacokinetic, *$RAUC_{0–12}$* accumulation ratio for $AUC_{0–12}$ (day 3/day 1), *RC_{max}* accumulation ratio for C_{max} (day 3/day 1), *SD* standard deviation, *t_{max}* time to reach C_{max}

Supplementary Table S5. Geometric mean and 90% CIs for ratio of day 3 to day 1 to assess adavosertib accumulation (PK analysis set)

	Day 1		Day 3		Point estimate, %	90% CI, %
	n	LS mean	n	LS mean		
$AUC_{0–12}$, nM·h	17	4791.0	15	11,060.0	230.8	200.3, 265.8
C_{max}, nM	18	714.6	14	1477.0	206.7	170.5, 250.5

Results are based on a linear mixed-effects model with a fixed effect for day and a random effect for patient. The point estimate and 90% CI were of geometric mean accumulation ratio of day 3 to day 1. Treatment: adavosertib 225 mg (3 x 75 mg capsules) bid on days 1–2 and qd on day 3. Accumulation ratio: $AUC_{0–12}$ and C_{max} on day 3 versus $AUC_{0–12}$ and C_{max} on day 1, respectively

$AUC_{0–12}$ area under the plasma concentration–time curve from 0 to 12 hours, *CI* confidence interval, *C_{max}* maximum plasma drug concentration, *LS* least squares, *PK* pharmacokinetic

Supplementary Table S6. Causally related adverse events (safety analysis set)

Preferred term	Number of patients (%)	
	**Adavosertib[a] AEs of any grade (*n* = 21)**	**Adavosertib[a] grade ≥3 AEs (*n* = 21)**
**Any AE[b]**	**11 (52.4)**	**2 (9.5)**
Nausea	6 (28.6)	1 (4.8)
Diarrhea	6 (28.6)	1 (4.8)
Vomiting	2 (9.5)	1 (4.8)
Anemia	1 (4.8)	1 (4.8)
Constipation	1 (4.8)	0
Decreased appetite	1 (4.8)	0

[a]Adavosertib 225 mg (3 x 75 mg capsules) bid on days 1–2 and qd on day 3

[b]Some patients experienced multiple AEs (as listed)

AE adverse event, *bid* twice daily, *qd* once daily
